# Supplementary material for: The interactome of intact mitochondria by cross-linking mass spectrometry provides evidence for coexisting respiratory supercomplexes
Source: Mol Cell Proteomics. 2017 Dec 8;17(2):216–32. doi: 10.1074/mcp.RA117.000470 (PMC5795388; doi:10.1074/mcp.RA117.000470)
Supplement: Supplemental Data [file supp_RA117.000470_133922_0_supp_23978_fzffwf.zip › spectra_annotation/mito_DR_spectra_annotation/26-1-44-1-14-1.pdf]

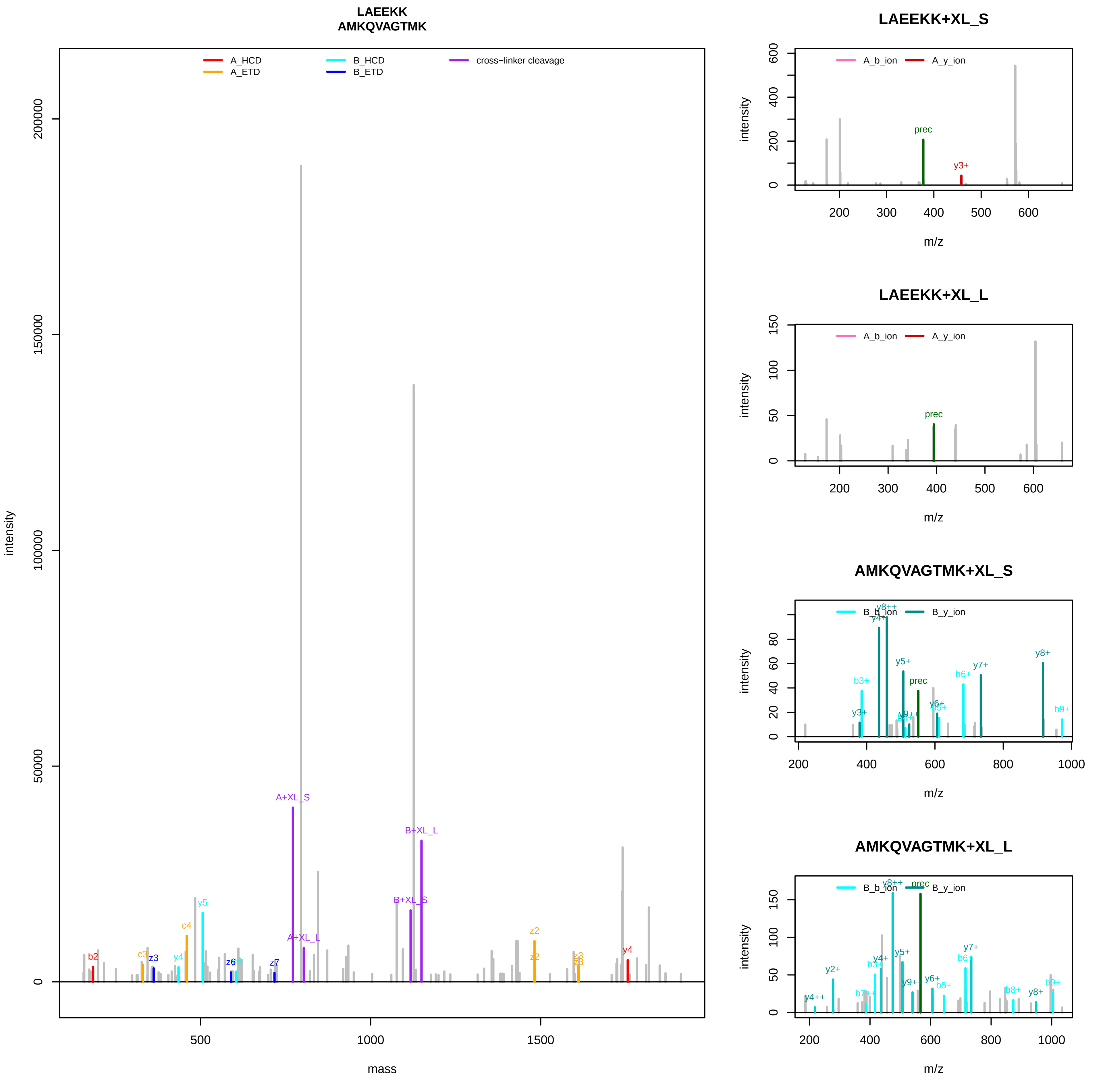
LAEEKK
AMKQVAGTMK
A_HCD
A_ETD
B_HCD
B_ETD
cross−linker cleavage
intensity
mass
b2
c3
z3
y4
c4
y5
z6
z7
A+XL_S
A+XL_L
B+XL_S
B+XL_L
z2
z3
y4
LAEEKK+XL_S
A_b_ion
A_y_ion
prec
y3+
m/z
LAEEKK+XL_L
A_b_ion
A_y_ion
prec
m/z
AMKQVAGTMK+XL_S
B_b_ion
B_y_ion
y8++
y4++
y5+
b3+
prec
b6+
y7+
y8+
y3+
y6+
b5+
b9+
m/z
AMKQVAGTMK+XL_L
B_b_ion
B_y_ion
y8++
prec
y2+
y4++
b3+
y4+
y5+
b7++
y9++
y6+
b5+
b6+
y7+
b8+
y8+
b9+
m/z
